# Structure–Activity Relationships and Molecular Docking Analysis of Mcl-1 Targeting Renieramycin T Analogues in Patient-derived Lung Cancer Cells

**DOI:** 10.3390/cancers12040875

**Published:** 2020-04-03

**Authors:** Korrakod Petsri, Masashi Yokoya, Sucharat Tungsukruthai, Thanyada Rungrotmongkol, Bodee Nutho, Chanida Vinayanuwattikun, Naoki Saito, Matsubara Takehiro, Ryo Sato, Pithi Chanvorachote

**Affiliations:** 1 Cell-based Drug and Health Products Development Research Unit, Chulalongkorn University, Bangkok 10330, Thailand; korrakod.petsri@gmail.com (K.P.); sucharat.tungsukruthai@gmail.com (S.T.); 2 Doctor of Philosophy Program in Interdisciplinary Pharmacology, Graduate School, Chulalongkorn University, Bangkok 10330, Thailand; 3 Department of Pharmacology and Physiology, Faculty of Pharmaceutical Sciences, Chulalongkorn University, Bangkok 10330, Thailand; 4 Graduate School of Pharmaceutical Sciences, Meiji Pharmaceutical University, 2-522-1 Noshio, Kiyose, Tokyo 204-8588, Japan; yokoya@my-pharm.ac.jp (M.Y.); naoki@my-pharm.ac.jp (N.S.); takehiro.matsubara@yof-linda.co.jp (M.T.); m186215@std.my-pharm.ac.jp (R.S.); 5 Structural and Computational Biology Research Unit, Department of Biochemistry, Faculty of Science, Chulalongkorn University, Bangkok 10330, Thailand; thanyada.r@chula.ac.th; 6 Program in Bioinformatics and Computational Biology, Faculty of Science, Chulalongkorn University, Bangkok 10330, Thailand; 7 Center of Excellence in Computational Chemistry (CECC), Department of Chemistry, Faculty of Science, Chulalongkorn University, Bangkok 10330, Thailand; b.nutho@gmail.com; 8 Division of Medical Oncology, Department of Medicine, Faculty of Medicine, Chulalongkorn University, Bangkok 10330, Thailand; chanida.vi@chula.ac.th

**Keywords:** renieramycin T, Structure–Activity Relationship, patient-derived primary lung cancer cells, lung cancer, apoptosis, Mcl-1, Molecular Docking Analysis

## Abstract

Myeloid cell leukemia 1 (Mcl-1) and B-cell lymphoma 2 (Bcl-2) proteins are promising targets for cancer therapy. Here, we investigated the structure–activity relationships (SARs) and performed molecular docking analysis of renieramycin T (RT) and its analogues and identified the critical functional groups of Mcl-1 targeting. RT have a potent anti-cancer activity against several lung cancer cells and drug-resistant primary cancer cells. RT mediated apoptosis through Mcl-1 suppression and it also reduced the level of Bcl-2 in primary cells. For SAR study, five analogues of RT were synthesized and tested for their anti-cancer and Mcl-1- and Bcl-2-targeting effects. Only two of them (TM-(–)-18 and TM-(–)-4a) exerted anti-cancer activities with the loss of Mcl-1 and partly reduced Bcl-2, while the other analogues had no such effects. Specific cyanide and benzene ring parts of RT’s structure were identified to be critical for its Mcl-1-targeting activity. Computational molecular docking indicated that RT, TM-(–)-18, and TM-(–)-4a bound to Mcl-1 with high affinity, whereas TM-(–)-45, a compound with a benzene ring but no cyanide for comparison, showed the lowest binding affinity. As Mcl-1 helps cancer cells evading apoptosis, these data encourage further development of RT compounds as well as the design of novel drugs for treating Mcl-1-driven cancers.

## 1. Introduction

Estimates of the worldwide cancer records of incidence and mortality from all types of cancers have revealed that lung cancer is the most common cause of cancer death, and is showing an increasing incidence. Furthermore, the 5-year survival rate from lung cancer is critically low due to its resistance to cancer therapy and due to disease relapse [1,2]. The improved clinical response as a result of targeted therapies has suggested that the use of more precise drugs focused on the molecular targets underlying the aggressiveness of the cancer and its drug resistance would be highly beneficial. 

It has been widely shown that one of the important hallmarks of cancer is the ability of the tumor cells to evade apoptosis [3]. In general, programmed apoptosis is a well-controlled cell death mechanism for the removal of unwanted or harmful cells. However, what frequently occurs in cancer is the upregulation of the anti-apoptotic B-cell lymphoma 2 (Bcl-2) family of proteins [4], which consequently facilitates oncogenesis through cell death resistance [5]. The Bcl-2 family of proteins belong to the class of BH-domain-containing proteins [6], which can be classified into anti-apoptotic members (Bcl-2, Mcl-1, Bcl-w, Bcl-xl, and Bfl-1/A1) and pro-apoptotic members (Bax, Bak, Bad, Bid, Bak, Bim, Puma, Noxa, Hrk, and Bmf). The anti-apoptotic proteins interact and are antagonized specifically to certain BH3-only proteins and pro-apoptotic proteins [7]. The pro-apoptotic proteins Bax and Bak are the key members that trigger permeabilization of the mitochondrial membrane and the release of pro-apoptogenic proteins. Recent evidence has suggested that the ability to evade apoptosis, survival during metastasis, and resistance to therapy are likely to be dependent on the expression and function of the pro-survival Bcl-2 proteins [8]. Consequently, it is not unexpected that the amplification and overexpression of pro-survival Bcl-2 proteins, such as Bcl-2 and Mcl-1, are found in many cancer types (e.g., non-small-cell lung cancer, breast cancer, ovarian cancer, prostate cancer, and pancreatic cancer) [9,10,11]. Bcl-2 family members, like Bcl-2 and Mcl-1, have been shown to dominate negative regulation for apoptosis control and to be responsible for chemotherapeutic resistance [12]. An analysis of cancer specimens revealed that up to 50% of lung cancer specimens exhibited elevated levels of Mcl-1 expression, which is associated with a poor prognosis for lung cancer patients [13,14]. Not unpredictably, the downregulation of Mcl-1 increases cancer cell sensitivity to standard anticancer drugs, such as etoposide, doxorubicin, and ABT-737 [14]. Moreover, the reduction of Mcl-1 sensitized osteosarcoma cells to chemotherapeutic agents [15]. In lung cancer, evidence from a microarray analysis indicated that Mcl-1 is highly expressed in the cancer tissue of non-small cell lung cancer (NSCLC) patients [16,17]. In addition, it was shown that NSCLC cell lines exhibited high levels of Mcl-1, and the inhibition of such a protein by siRNA could potently mediate NSCLC cell apoptosis [18]. Additionally, evidence has suggested that, in NSCLC, Mcl-1 inhibition showed superior potential for having a cancer therapeutic effect when compared with Bcl-xl inhibition [18]. Taken together, new drugs with modes of action involving eliminating Mcl-1 in lung cancer cells are of interest as candidates for Mcl-1-targeted therapy.

Marine organisms function as a reservoir of potent marine-derived agents capable of inhibiting the growth of cancer cells, as has been demonstrated in in vitro and in vivo studies [19,20]. For instance, there are four marine agents approved for use in the treatment of cancers: cytarabine, trabectedin, eribulin mesylate, and the conjugated antibody brentuximab vedotin [21]. Moreover, it has been found that substances from marine organisms, such as renieramycins, have the potential to prevent tumor formation and induce cell death via the apoptosis pathway [22]. A previous study reported that renieramycin T (RT), a renieramycin-related compound isolated from the blue sponge *Xestospongia* sp., was dominantly toxic to lung cancer cells and mainly exerted this effect through apoptosis induction via the targeting of Mcl-1 for ubiquitin-proteasomal degradation [23]. As RT has a complex structure composed of several chemical moieties, understanding the structure–activity relationships (SARs) is a necessity for identification of the active moieties that are critical for drug action and that hold promise to increase drug precision and potency. Using RT as a lead compound, we aimed to establish such structure–activity relationships (SARs) and the subsequent SAR-directed optimization for treatment. The newly synthesized simplified parts of RT were developed and the active parts as well as the required moieties of the compound for the Mcl-1-targeted effect were evaluated in the present study utilizing protein analysis in combination with molecular docking simulation.

## 2. Results

### 2.1. Cytotoxicity and Apoptosis-inducing Effect of RT on Patient-derived Primary Lung Cancer Cells

**Figure 1:**
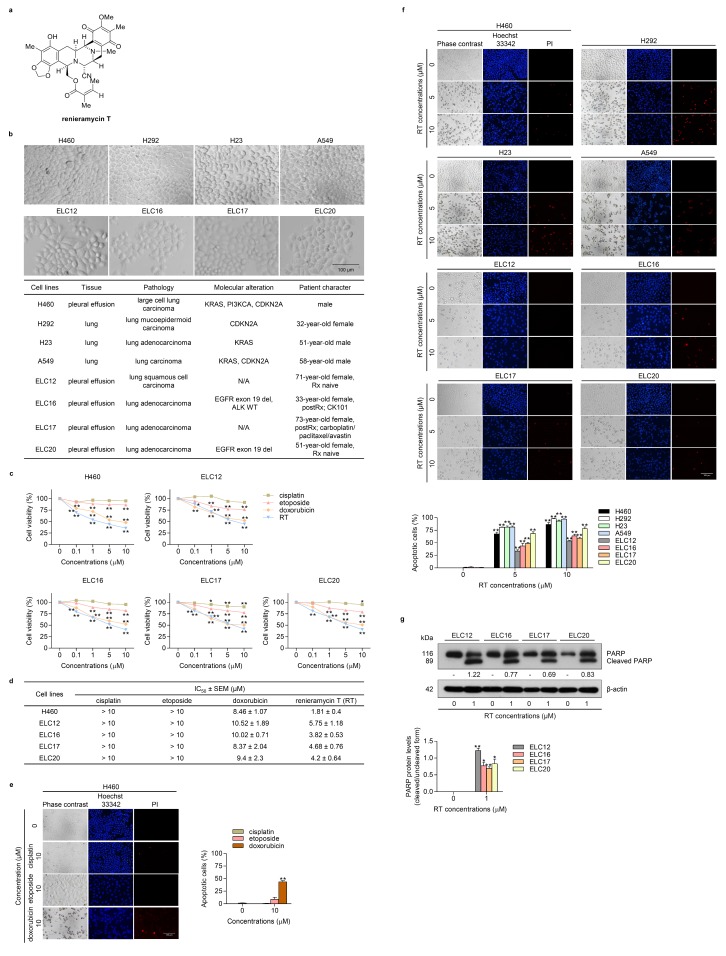
Effects of renieramycin T (RT) on cell viability and apoptotic cell death in non-small cell lung cancer (NSCLC) cell lines (H460, H292, H23, and A549) and patient-derived primary cancer cell lines (ELC12, ELC16, ELC17, and ELC20). (**a**) The structure of RT. (**b**) The morphology of NSCLC and patient-derived primary cancer cell lines and their molecular characteristics. (**c**) H460, ELC12, ELC16, ELC17, and ELC20 cells were seeded and treated with 0–25 μM of RT or chemotherapeutic drugs (cisplatin, etoposide, and doxorubicin) for 24 h. Then, the MTT assay was performed to determine the percentages of cell viability. (**d**) The IC_50_ in all cells was calculated in comparison to the untreated control. (**e–f**) Cells were seeded and treated with 0–10 μM of RT or chemotherapeutic drugs (cisplatin, etoposide, and doxorubicin) for 24 h before adding Hoechst 33342 and PI to stain the cell nucleuses. Images were detected by using a fluorescence microscope and the percentages of nuclear-fragmented and propidium iodide (PI)-positive cells were calculated. (**g**) ELC12, ELC16, ELC17, and ELC20 cells were treated with 0–1 μM of RT for 24 h. Western blot analysis was performed to detect the PARP and cleaved PARP protein levels. The blots were reprobed with β-actin to confirm an equal loading of each of the protein samples and densitometry was used to calculate the protein expression levels. Densitometric values of protein levels were presented as the fold changes relative to uncleaved form of the protein. Data represent the mean ± SEM (*n* = 3) (* 0.01 ≤ *p* < 0.05, ** *p* < 0.01, compared with the untreated control). Gene symbols: KRAS (Kirsten Rat Sarcoma Viral Oncogene Homolog), PI3KCA (Phosphatidylinositol-4, 5-Bisphosphate 3-Kinase Catalytic Subunit Alpha), CDKN2A (Cyclin Dependent Kinase Inhibitor 2A), EGFR (Epidermal Growth Factor Receptor), ALK (Anaplastic Lymphoma Receptor Tyrosine Kinase). Abbreviations: N/A (not available), del (deletion), WT (wild type), Rx (treatment).

Chemotherapeutic drug resistance is accepted to be a major cause of therapeutic failure, tumor recurrence, and disease progression in lung cancer [24]. Mcl-1, an anti-apoptotic member of the Bcl-2 family, was demonstrated to be mainly involved in chemotherapeutic resistance as this protein is frequently found to be highly expressed in lung cancer [25] and the diminishment of Mcl-1 can lead to cancer cell death [26,27]. To characterize the potency of the anti-cancer activity of RT (Figure 1a), we determined the cytotoxic profile of RT in chemotherapeutic resistant primary lung cancer cells (ELC12, ELC16, ELC17, and ELC20) and lung cancer cell lines (H460). The basic cell morphology of the NSCLC and patient-derived primary cancer cell lines and the molecular characteristics are shown in Figure 1b. The results indicated that RT exerted a superior cytotoxic potency when compared with the commonly used chemotherapeutic drugs, including cisplatin, etoposide, and doxorubicin, at the equivalent concentrations (Figure 1c). Figure 1c shows that nearly all of the lung cancer cells were resistant to cisplatin at 0–10 µM, as the cell viability was found to be above 90% after treatment, while doxorubicin and RT showed comparable potent cytotoxic effects and both compounds could reduce cancer cell viability by approximately 70% at the 10 µM concentration. The half maximal inhibitory concentrations (IC_50_) values of RT and the commercial drugs were calculated and the results indicated that the IC_50_ of RT was generally lower than that of the chemotherapeutic drugs. Importantly, RT showed greater potency compared to that of doxorubicin in all the cells (Figure 1d). The apoptotic cell death and necrosis were further evaluated by Hoechst33342 and propidium iodide (PI) staining, respectively. We tested the apoptosis induction effect of cisplatin, etoposide, and doxorubicin in H460 cells and found consistent results with the cytotoxicity results, showing that doxorubicin caused the highest apoptosis, as indicated by the fragmented or condensed nuclei (Figure 1e). Then, the apoptosis induction effect of RT was evaluated in all lung cancer cells (H460, H292, H23, A549, ELC12, ELC16, ELC, 17, and ELC 20). The result revealed that RT caused an increase in apoptosis in a concentration-dependent manner, whereas it exhibited a minimal necrotic cell death effect, as shown in Figure 1e,f. We confirmed the apoptotic cell death by determination of cleaved PARP protein using Western blot analysis. The result showed an increase of cleaved PARP in response to RT treatment compared to control (Figure 1g). 

### 2.2. Cytotoxic and Mcl-1-targeting Activities of the Simplified Right-half model of RT Compounds

**Figure 2:**
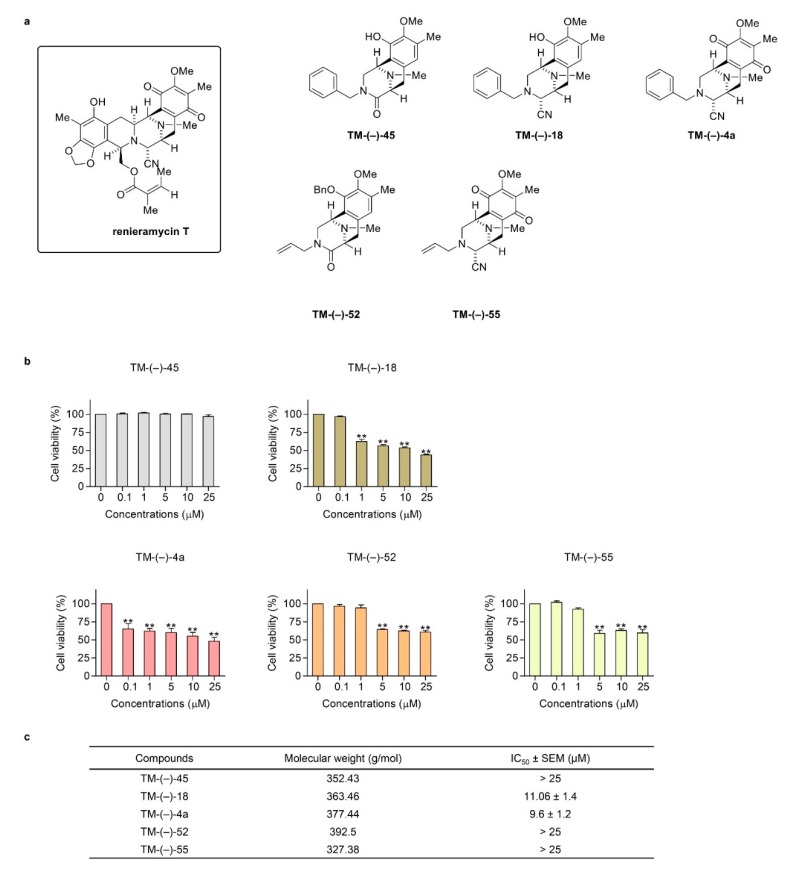
Cytotoxic effects of the simplified right-half model of RT compounds on the NSCLC cell line (H460). (**a**) The structures of RT and the simplified right-half model of RT compounds: TM-(–)-45, TM-(–)-18, TM-(–)-4a, TM-(–)-52, and TM-(–)-55. (**b**) The H460 cell line was treated with 0–25 μM of the compounds for 24 h. Then, the MTT assay was used to determine the percentages of cell viability. (**c**) IC_50_ values in each cell line were calculated in comparison to the untreated control. Data represent the mean ± SEM (*n* = 3) (* 0.01 ≤ *p* < 0.05, ** *p* < 0.01, compared with the untreated control).

The SAR of a bioactive compound is very useful information that could facilitate the development of drugs, as this information describes the critical parts to be exploited for drug action as well as helps to increase the potency of the initially detected activity. As RT has been shown to have promising anti-cancer activity against lung cancer through its Mcl-1-targeted activity, as demonstrated in our previous study [23], we designated RT as a lead compound and generated simplified right-half models in order to elucidate the SAR. The structures of RT and the simplified right-half model of RT compounds, namely TM-(–)-45, TM-(–)-18, TM-(–)-4a, TM-(–)-52, and TM-(–)-55, are shown in Figure 2a. The synthesized compounds with various modifications were then tested for cytotoxicity in H460 cells. The results obtained from the MTT assay showed that TM-(–)-18, TM-(–)-4a, TM-(–)-52, and TM-(–)-55 significantly decreased the cell viability of the lung cells, whereas TM-(–)-45 had no effect (Figure 2b). Moreover, the IC_50_ values of the simplified right-half model of RT compounds were determined, and the results suggested that TM-(–)-18 and TM-(–)-4a retained the cytotoxicity of RT (Figure 2c). 

**Figure 3:**
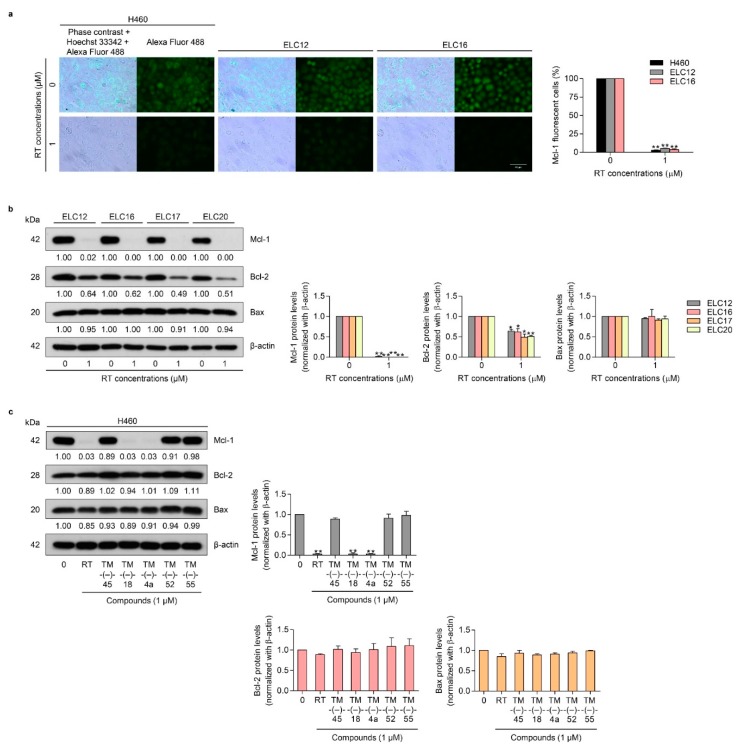
Effects of RT on the expression of the apoptotic-related proteins: Mcl-1, Bcl-2, and Bax in the NSCLC cell line (H460) and patient-derived primary cancer cell lines (ELC12, ELC16, ELC17, and ELC20). (**a**) H460, ELC12, and ELC16 cell lines were seeded and treated with 0–1 μM of RT for 24 h. Then, immunofluorescence analysis was performed using an antibody against Mcl-1. The Alexa Fluor 488 conjugated secondary antibody and Hoechst 33342 were added and the images were visualized under a fluorescence microscope. (**b**) ELC12, ELC16, ELC17, and ELC20 cell lines were seeded and treated with 0–1 μM of RT for 24 h. Western blot analysis was performed to detect the Mcl-1, Bcl-2, and Bax protein levels. The blots were reprobed with β-actin to confirm an equal loading of each of the protein samples and densitometry was used to calculate the relative protein levels. Densitometric values of protein expression levels were presented as the fold changes relative to β-actin. (**c**) H460 cells were seeded and treated with 0–1 μM of RT, TM-(–)-45, TM-(–)-18, TM-(–)-4a, TM-(–)-52, and TM-(–)-55 for 24 h. After that, Western blot analysis was performed to detect the Mcl-1, Bcl-2, and Bax protein levels. Data represent the mean ± SEM (*n* = 3) (* 0.01 ≤ *p* < 0.05, ** *p* < 0.01, compared with the untreated control).

In order to verify the Mcl-1-targeted activity of the simplified right-half compounds of RT, we first confirmed the Mcl-1-targeted action of RT in primary lung cancer cells. We determined the Mcl-1 level in H460, ELC12, and ELC16 by immunofluorescence assay. The results showed that RT reduced the fluorescence intensity, reflecting the Mcl-1 protein, in H460, ELC12, and ELC16 cells (Figure 3a). Western blot analysis was used to confirm the effect of RT on Mcl-1 and its possible effect on the anti-apoptotic Bcl-2 proteins. The Mcl-1, Bcl-2, and proapoptotic Bax in the Bcl-2 family proteins were determined and the results showed that RT significantly decreased the level of Mcl-1 and Bcl-2 in primary lung cancer cells. In contrast, RT had no effect on the Bax protein (Figure 3b). It was noteworthy that while we showed in this study that RT reduced both Mcl-1 and Bcl-2 in primary lung cancer cells, RT was previously shown to have a minimal effect on Bcl-2 in H460 cells [28].

Next, we examined whether the simplified right-half model of RT could target the levels of Mcl-1, Bcl-2, and Bax. Consequently, H460 cells were treated with all the compounds of the RT right-half models and it was found that only TM-(–)-18 and TM-(–)-4a significantly decreased the level of Mcl-1 in H460 cells (Figure 3c). Together with the results of the cytotoxicity tests in Figure 2b,c that indicate that only TM-(–)-18 and TM-(–)-4a exerted significant cytotoxicity, it is very likely that the cytotoxic mode of action of TM-(–)-18 and TM-(–)-4a depended on their Mcl-1-targeting activity.

### 2.3. TM-(–)-18 and TM-(–)-4a compounds Induced Apoptotic Cell Death in Patient-derived Primary Cancer Cell Lines Through the Suppression of Mcl-1 and Bcl-2

**Figure 4:**
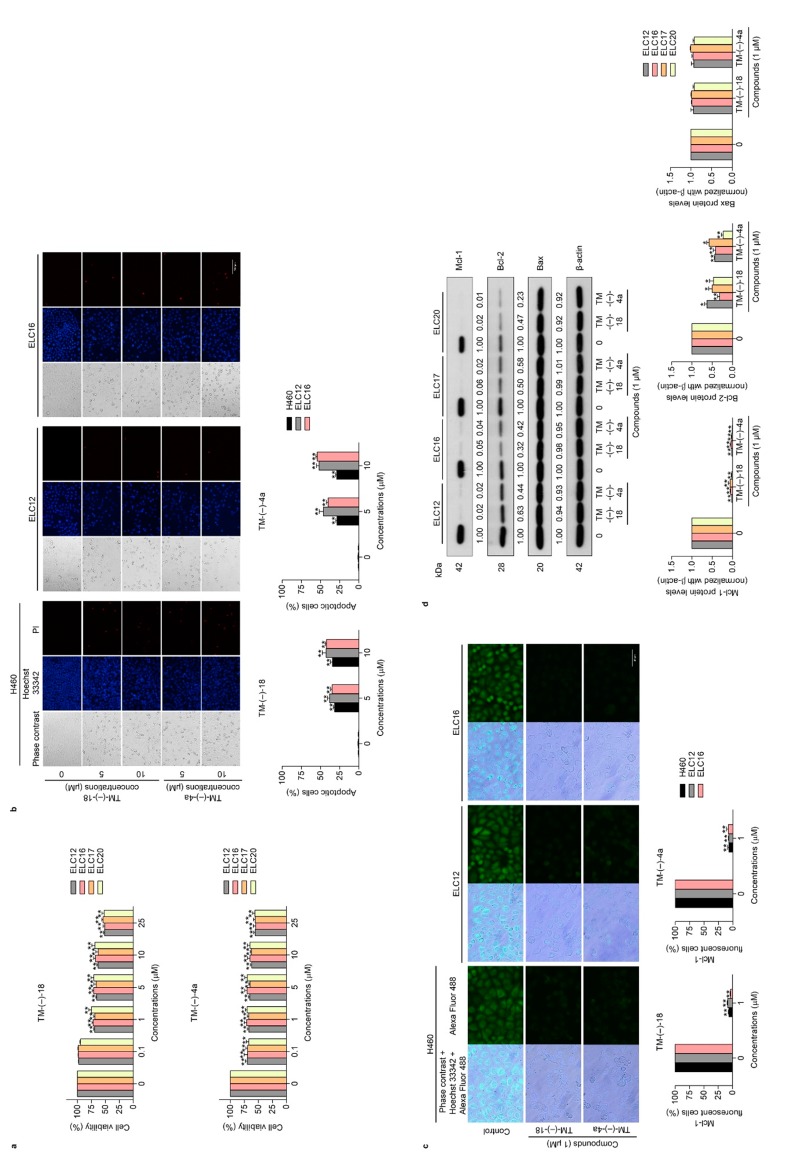
Effects of the simplified right-half model of RT compounds: TM-(–)-18 and TM-(–)-4a on cell viability, apoptotic induction, and apoptotic-related protein expressions in patient-derived primary cancer cell lines (ELC12, ELC16, ELC17, and ELC20). (**a**) ELC12, ELC16, ELC17, and ELC20 cells were seeded and treated with 0–25 μM of TM-(–)-18 and TM-(–)-4a for 24 h. Then, the MTT assay was performed to determine the percentages of cell viability. (**b**) H460, ELC12, and ELC16 cells were seeded and treated with 0–10 μM of TM-(–)-18 and TM-(–)-4a for 24 h. Hoechst 33342 and PI were added and then the images were visualized using a fluorescence microscope. (**c**) H460, ELC12, and ELC16 cells were seeded and treated with 0–1 μM of TM-(–)-18 and TM-(–)-4a for 24 h before performing immunofluorescence using the Mcl-1 primary antibody. Alexa Fluor 488 conjugated secondary antibody and Hoechst 33342 were added and the images were visualized under a fluorescence microscope. (**d**) ELC12, ELC16, ELC17, and ELC20 cell lines were seeded and treated with 0–1 μM of TM-(–)-18 and TM-(–)-4a for 24 h. Western blot analysis was performed to detect the Mcl-1, Bcl-2, and Bax protein levels. The blots were reprobed with β-actin to confirm an equal loading of each of the protein samples and densitometry was used to calculate the relative protein levels. Densitometric values of protein expression levels were presented as the fold changes relative to β-actin. Data represent the mean ± SEM (*n* = 3) (* 0.01 ≤ *p* < 0.05, ** *p* < 0.01, compared with the untreated control).

Having shown the potential Mcl-1 suppression in H460 cells, we next confirmed the Mcl-1- and Bcl-2-targeting activity of TM-(–)-18 and TM-(–)-4a in primary lung cancer cells. ELC12, ELC16, ELC17, and ELC20 cells were treated with 0–25 μM of TM-(–)-18 and TM-(–)-4a for 24 h, and cell viability, apoptosis induction, and the Mcl-1, Bcl-2, and Bax levels were determined. The results showed that TM-(–)-18 and TM-(–)-4a decreased cell viability in all the cell lines (Figure 4a). Next, the morphology of the apoptotic cell death was evaluated by Hoechst33342 and PI double staining. Patient-derived primary cancer cells were treated with TM-(–)-18 and TM-(–)-4a at various concentrations for 24 h, and then the cells were co-stained with Hoechst 33342 and PI. The results revealed that TM-(–)-18 and TM-(–)-4a increased apoptotic cells in a concentration-dependent manner, as shown in Figure 4b and Figure S3a. Immunofluorescence detecting of the Mcl-1 protein in the H460, ELC12, and ELC16 cell lines in response to TM-(–)-18 and TM-(–)-4a treatment showed that TM-(–)-18 and TM-(–)-4a significantly reduced the Mcl-1 fluorescence intensity in all the cells (Figure 4c). Consistently, the protein analysis of Mcl-1, Bcl-2, and Bax indicated that treatment of the cells with TM-(–)-18 and TM-(–)-4a caused a dramatic reduction in the Mcl-1 protein in all the primary lung cancer cells (Figure 4d). The Bcl-2 protein was found to significantly decrease in a concentration-dependent manner, while the treatment had no effect on the Bax protein (Figure 4d). Taken together, this result implied that the mechanism of action of RT and the simplified right-half model of RT compounds (TM-(–)-18 and TM-(–)-4a) involved the targeting of the Mcl-1 protein and, at least in part, Bcl-2 suppression.

### 2.4. Molecular Docking Simulation Revealed the RT, TM-(–)-18, and TM-(–)-4a Interactions with the Mcl-1 Protein

**Figure 5:**
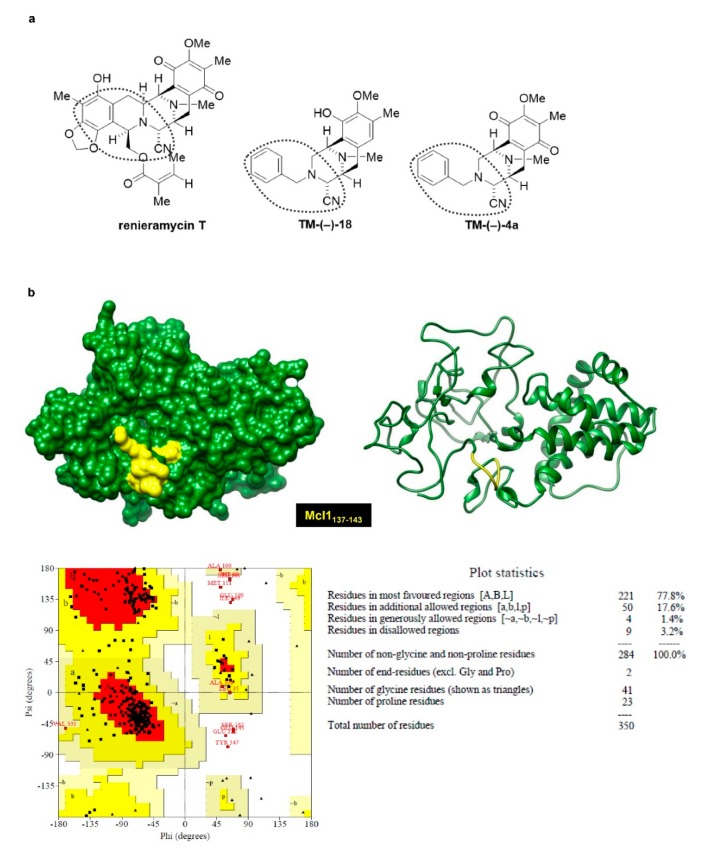
(**a**) Structures of RT, TM-(–)-18, and TM-(–)-4a. Circled part represents a similar structure between these three compounds. (**b**) Stereochemical quality of the homology model of Mcl-1 created by the I-TASSER server. Ramachandran plot of Mcl-1 generated by PROCHECK. Areas colored by red, yellow, beige, and white indicate the most favored, additionally allowed, generously allowed, and disallowed regions, respectively.

**Figure 6:**
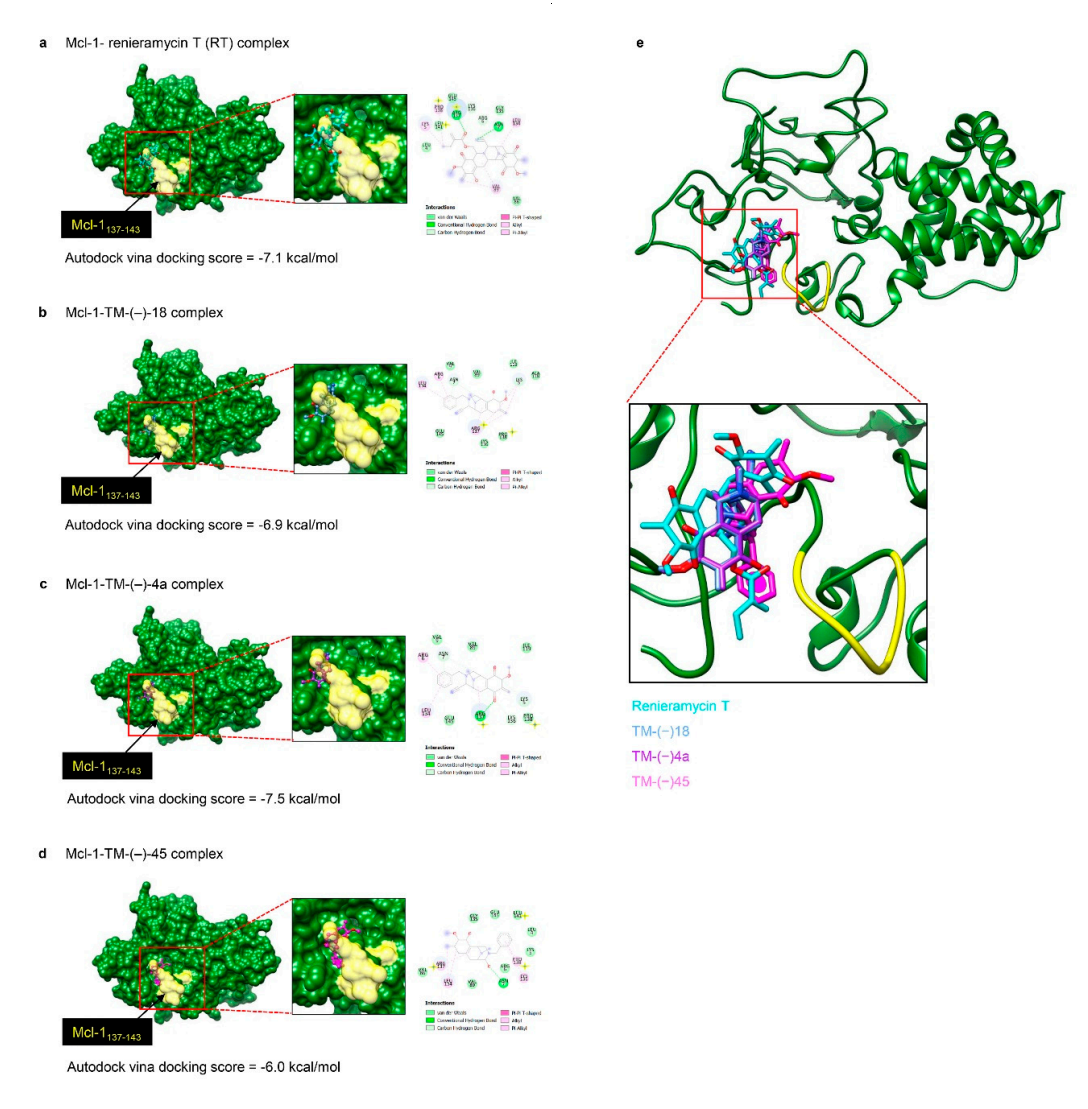
Binding mode and docking energy of: (**a**) renieramycin T, (**b**) TM-(−)-18, (**c**) TM-(−)-4a, and (**d**) TM-(−)-45 bound to the binding site of Mcl-1 (residues 137–143) taken from the AutoDock Vina molecular docking study. (**e**) Superimposition structure of each compound at the binding site of Mcl-1.

Regarding SAR, we compared the chemical structures of the active (TM-(–)-18 and TM-(–)-4a) and non-active (TM-(–)-45, TM-(–)-52, and TM-(–)-55) compounds of the RT simplified right-half model and found that those in the circled part in Figure 5a could be the important groups for Mcl-1 suppression, as this part has a similar structure among RT, TM-(–)-18, and TM-(–)-4a, but not in the inactive compounds. To confirm the interaction between RT, TM-(–)-18, and TM-(–)-4a and the suspected moieties on the Mcl-1-targeting action, molecular docking simulation was applied. Moreover, the relative binding energies of the three simplified right-half model of RT compounds (TM-(−)-18, TM-(−)-4a, and TM-(−)-45) in comparison to the prototype compound (RT) were estimated and insights into how these ligands bind to the binding site of Mcl-1 were gained. It should be noted that the relationship between the binding energy derived from the docking calculations and the binding affinity was associated with the concept of, ‘The higher the binding affinity of the compound, the lower the docking energy’ [29]. The docking results showed that the binding affinity of TM-(−)-18 and TM-(−)-4a to Mcl-1 was in a similar range to that of RT (binding energies of −6.9, −7.5, and −7.1 kcal/mol for TM-(−)-18, TM-(−)-4a, and RT, respectively) (Figure 6a–c,e). This prediction reflected that these three compounds could bind well to the binding site of Mcl-1, as previously supported by the experimental data. Nonetheless, the binding energy of TM-(−)-45 (−6.0 kcal/mol) was somewhat higher than those of the other compounds (Figure 6d), indicating that this compound exhibited the lowest binding affinity toward Mcl-1, leading to its incapability to induce Mcl-1 depletion. This was possibly due to the distinct orientation of TM-(−)-45 within the binding site of Mcl-1, in which both its cyanide and benzene ring (circled part in Figure 5a) moieties pointed outward to the protein binding pocket, thus creating weaker interactions with the critical residues (Arg137, Pro138, Ala139, Val140, Leu141, and Pro142).

## 3. Discussion

In cancer biology, it is well known that the evasion of apoptosis is a prominent hallmark of cancer [3]. Apoptosis is an important component of various processes as it contributes to the elimination of unwanted cells to maintain the equivalence between cell survival and cell death. Therefore, the dysregulation of apoptosis results in sustained cell proliferation and enhanced tumor development [30], and consequently, drugs or treatment strategies that can restore the apoptotic signaling pathways may benefit the management of cancer. 

The essential regulators of apoptosis are the Bcl-2 family of proteins [31]. Regarding evading apoptosis, the roles of anti-apoptotic members, like Mcl-1 and Bcl-2, have been shown in many in vitro and in vivo studies [5,15,32,33,34,35,36]. The protein levels of Bcl-2 and Mcl-1 in various types of tumor tissues were found to be frequently augmented at a higher ratio than the other anti-apoptotic proteins in the Bcl-2 family [26]. In particular, increased expressions of Bcl-2 and Mcl-1 reflect a poor prognosis for many malignancies, including lung cancer [37,38,39]. Not only is their increased expression critical for oncogenesis and cancer progression, but these proteins are also involved in conferring chemotherapeutic drug resistance [35,40,41,42,43]. Research was performed using Bcl-2 as a target for overcoming chemoresistance through BCL2 gene silencing to improve the clinical outcome in small-cell lung cancer [44]. Furthermore, in a mouse lung adenocarcinoma model, Mcl-1 overexpression was shown to help tumor progression by inhibiting Myc-induced apoptosis [40]. Taken together, compounds with potent activity for eliminating Bcl-2 or Mcl-1 in cancer cells are of great interest as good candidates for targeted therapy. In agreement with the use of Mcl-1 as a target for cancer therapy [11], our previous study highly supported this concept, where in our experiments, the treatment of RT in an NSCLC cell line (H460) resulted in apoptotic cell death through an Mcl-1 proteasomal degraded mechanism [23]. In the same way, not only in a known lung cancer cell line, but we also proved that the absence of Mcl-1 after RT treatment in primary lung cancer cell lines derived from patients (Figure 3a,b) could trigger the apoptotic pathway, which led to the death of cancer cells (Figure 1f,g). 

Renieramycin T (RT) (Figure 1a) is a tetrahydroisoquinoline alkaloid compound that is part of the renieramycin family and was first isolated from the blue sponge *Xestospongia* sp. by pretreatment with potassium cyanide in a study in 2009 [45]. Recently, its anti-cancer activities have been reported against several types of cancer cells, such as colon (HCT116), prostate (DU145) [46], non-small cell lung (H292, H460, and QG56) [47], breast (T47D), and pancreatic (AsPC1) cancer cells [45]. Moreover, a modified form of RT, 5-*O*-acetyl-renieramycin T, was shown to induce the death of lung cancer stem cells and sensitize cisplatin-mediated apoptosis in lung cancer cells [48]. Our previous study revealed that the effects of RT on the apoptotic mechanism depended on the disappearance of Mcl-1 through the increase in Mcl-1 protein degradation [23]. In this study, we further confirmed that RT also had anti-cancer activities as well as Mcl-1-targeted activity in patient-derived primary lung cancer cells, with a lower IC_50_ compared to other first-line chemotherapeutic drugs (Figure 1b–g, Figure 3a,b). It was newly discovered that RT could decrease the level of the Bcl-2 protein in primary lung cancer cells, while we did not observe this effect before in the lung cancer cell line H460 [23]. The explanation for this may due to the specific mutations of H460 cells, including KRAS and PI3K. The mutation of these two oncogenes may cause a high expression of Bcl-2 in H460 cells [28]. Gathering all the collected information, it is not surprising that RT could be an outstanding applicant for further clinical investigation in anti-cancer drug development. 

In spite of the marvelous anti-cancer effects of RT, it is hard to deny that RT is difficult to fabricate in massive large-scale synthesis due to its large and complicated structure. Besides, its structure–activity relationships are still unrecognized. Therefore, simplified right-half model of RT compounds (TM-(–)-45, TM-(–)-18, TM-(–)-4a, TM-(–)-52, and TM-(–)-55) were established using RT as a prototype (Figure 2a) to help define which part of RT is necessary for achieving the Mcl-1-targeted effect. After screening by treatment against the H460 cell line, we found that only TM-(–)-18 and TM-(–)-4a exerted cytotoxic activities, with IC_50_ values less than 25 µM (Figure 2b,c). Moreover, when we performed Western blot analysis using a primary antibody against Mcl-1, the results revealed that treatment with TM-(–)-18 and TM-(–)-4a could diminish Mcl-1 protein levels in the H460 cell line compared to the untreated control and the other simplified right-half model of RT compounds (Figure 3c). Having compared the structures between the compounds causing Mcl-1 depletion, namely RT, TM-(–)-18, and TM-(–)-4a, and the compounds not causing Mcl-1 depletion, namely TM-(–)-45, TM-(–)-52, and TM-(–)-55, we identified two similar parts among the Mcl-1-depleted compounds that did not simultaneously appear in the others. Hence, we hypothesized that both cyanide and the benzene ring in the circled parts in Figure 5a were essential in the Mcl-1-depletion mechanism. Not only the H460 cell line, but we also examined the effects of TM-(–)-18 and TM-(–)-4a with patient-derived primary lung cancer cells. TM-(–)-18 and TM-(–)-4a demonstrated cytotoxic effects and apoptotic induction effects in both NSCLC (H460) and patient-derived primary lung cancer cells (Figure 4a,b and Figure S3a). Furthermore, the results from the TM-(–)-18 and TM-(–)-4a treatments showed a decrease in Mcl-1 and Bcl-2 protein levels in primary lung cancer cells (Figure 4c,d), but as we previously discussed in the section about the RT-treatment conditions, Mcl-1 depletion was the main mechanism by which these compounds induced apoptosis. 

It has been shown that the anti-apoptotic potency of the Bcl-2 family of proteins primarily relies on their stability [49]. Mcl-1 is considered a very unstable protein compared to the other Bcl-2 family proteins and the degradation of Mcl-1 can be induced by anti-cancer agents [50,51,52,53]. The stabilization and degradation of Mcl-1 were reported to be regulated by a phosphorylation mechanism at the PEST region, which is the N-terminus of Mcl-1 enriched in proline (P), glutamate (E), serine (S), and threonine (T) residues and rich in putative phosphorylation sites [54]. Several pieces of evidence have suggested that the phosphorylation of Mcl-1 induced by extracellular signal-regulated kinases (ERK), c-Jun NH2-terminal kinase (JNK), and p38 at Threonine 92, Threonine 163, and Serine 121 can stabilize the Mcl-1 protein level [55,56,57]; whereas, the phosphorylation by glycogen synthase kinase-3 (GSK-3) at Serine 155 and Serine 159 destabilizes the Mcl-1 protein level and inhibits the interaction between Mcl-1 and the pro-apoptotic protein Bim [58,59,60,61]. From this information, we made the hypothesis that, supposing our compounds attached Mcl-1 at ERK, JNK, or p38 binding sites, they might prevent the phosphorylation mechanism of those molecules and lead to destabilization of the Mcl-1 protein level. To prove our hypothesis, we applied computational molecular docking using the mitogen-activated protein kinase (MAPK) docking motif, also known as the D-motif, found in the Mcl-1 protein sequence 137–143 as the target for the compounds and Mcl-1 interactions to characterize the behavior of these small molecule compounds at the binding site of the Mcl-1 protein [62,63]. Likewise, the circled part of the compounds in Figure 5a, which it is supposed are necessary for their mechanisms, was deployed as a docking site for examination of the binding properties and SARs. After investigation (Figure 6a–e), the results revealed that the binding affinity of RT, TM-(–)-18, and TM-(–)-4a was high and in a similar range, which indicated their ability to bind properly with Mcl-1 and induce destabilization of the protein; whereas, the binding affinity of TM-(–)-45, which has a benzene ring but no cyanide, was lower than the others, thus reflecting its incapability to induce Mcl-1 destabilization, as previously supported by the experimental data. 

The appearance of both cyanide and a benzene ring (Figure 5a) in RT, TM-(–)-18, and TM-(–)-4a was found to be necessary for the induction of Mcl-1 destabilization. This study energetically supported the SAR concept, which is key to many aspects of new drug discovery [64] and provides beneficial information for further anti-cancer drug modification and drug development as SARs can then be used to predict the activities of new molecules from their molecular structure because there is a relationship between molecular structures and their biological activity. This allows the modification of the effect or the potency of a bioactive compound by changing its chemical structure. Therefore, the SAR concept is essential in drug discovery to guide the acquisition or synthesis of desirable new compounds, as well as to further characterize existing molecules.

**Figure 7:**
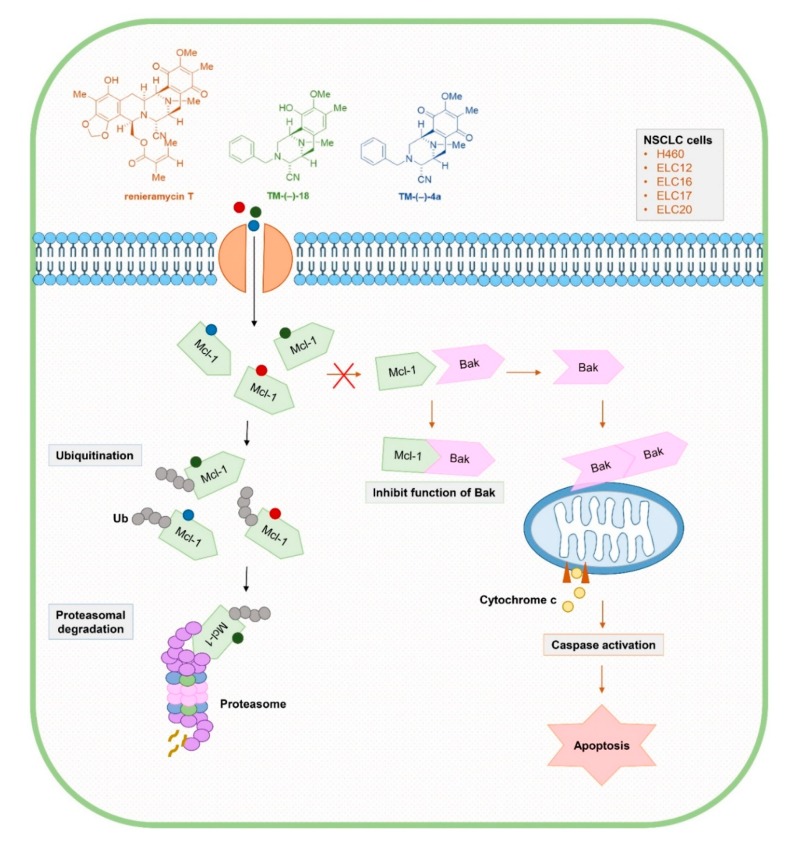
Renieramycin T (RT) and the simplified right-half model of RT compounds: TM-(–)-18 and TM-(–)-4a could enhance the absence of the Mcl-1 protein levels through Mcl-1 proteasomal degradation, resulting in the apoptosis of NSCLC cells. Mcl-1 normally functions as an anti-apoptotic protein by forming a complex with Bak. However, when Mcl-1 disappears, Bak is relieved to form an oligomerization that can permeabilize the outer membrane of mitochondria. Cytochrome c is released to initiate the apoptosis mechanism through caspase activation.

Our results suggested that RT, TM-(–)-18, and TM-(–)-4a exerted an apoptotic induction effect via the destabilization of the Mcl-1 protein (Figure 7). The interaction of the protein with the compounds promoted the ubiquitination and proteasomal degradation of Mcl-1 protein. As Mcl-1 has an important function to inhibit apoptosis by interacting and sequestering the pro-apoptotic Bak protein, the diminishing of Mcl-2 cause by RT and its analogues could facilitate the Bak dimerization. The decrease in cellular Mcl-1 protein or the disruption of Mcl-1-Bak interaction can lead to Bak oligomerization, mitochondrial membrane destabilization, cytochrome c release, and apoptosis [25,30]. 

## 4. Materials and Methods 

### 4.1. Non-small Cell Lung Cancer Cell Lines and Cultures

Non-small cell lung cancer cells used in the experiments including NCI-H460 [H460] (ATCC^®^ HTB-177™, RRID: CVCL_0459), NCI-H292 [H292] (ATCC^®^ CRL-1848™, RRID: CVCL_0455), NCI-H23 [H23] (ATCC^®^ CRL-5800™, RRID: CVCL_1547), and A549 (ATCC^®^ CCL-185™, RRID: CVCL_0023) were obtained from the American Type Culture Collection (Manassas, VA, USA). H460, H292, and H23 cells were grown in Roswell Park Memorial Institute (RPMI) 1640 medium, and A549 cells was grown in Dulbecco’s Modified Eagle Medium (DMEM). All cell culture mediums were supplemented with 10% fetal bovine serum (FBS), 2 mM L-glutamine, and 100 units/mL of each of penicillin and streptomycin. Cells were placed in a humidified atmosphere of 5% carbon dioxide (CO_2_) at 37 °C.

### 4.2. Patient-derived Primary Lung Cancer Cell Line Preparation from Malignant Pleural Effusion

The patient-derived malignant cancer cells were isolated from pleural effusions of recurrent or advanced stage non-small cell lung cancer patients who had been diagnosed at the King Chulalongkorn Memorial Hospital. The protocol of conduction was approved by the Ethics Committee of the Faculty of Medicine, Chulalongkorn University, Bangkok, Thailand (IRB 365/62) and was obtained informed consents from all participants. This study was carried out in accordance with the principles of World Medical Association Declaration of Helsinki. Primary cancer cells were collected from pleural effusion (500–1000 mL) through thoracentesis. The collected samples were centrifuged at 300 g for 10 min, at 4 °C and the cells were resuspended in RPMI medium with 10% FBS, 2 mM L-glutamine, and 100 units/mL of each of penicillin and streptomycin. After culturing for 10–15 passages, they were characterized as the patient-derived primary cancer cell lines (ELC12, ELC16, ELC17, and ELC20). The characteristics as well as their status of mutation were presented in the Figure 1b.

### 4.3. Renieramycin T (RT) Prepararation

From the previous study [23], renieramycin T (RT) was isolated from the Thai blue sponge *Xestospongia* sp. collected from Sichang Island, Chonburi Province, Thailand. After isolated process, RT was obtained in an orange amorphous solid form with the purity more than 99% and spectroscopic data matching to Daikuhara report [45]. The nuclear magnetic resonance (NMR) values were demonstrated in the previous report [23]. Before using in the experiments, RT was dissolved to 50 mM stock solution by dimethyl sulfoxide (DMSO), stored at -20 °C, and freshly diluted to desired concentrations with medium in each experiment. DMSO final concentration was less than 0.5% referring to non-toxicity for cancer cells.

### 4.4. Simplified Right-half model of RT Compounds Synthesis

**Figure 8:**
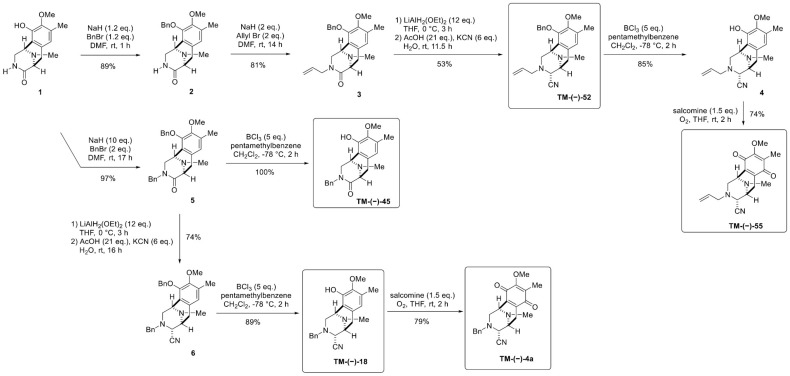
Simplified right-half model of RT compounds: TM-(–)-45, TM-(–)-18, TM-(–)-4a, TM-(–)-52, and TM-(–)-55 synthetic pathways. Compound **1** could be easily obtained from L-Tyr [65]. The phenol of compound **1** was selectively protected with 1.2 eq. of NaH and BnBr in DMF to give compound **2**. The alkylation of the lactam nitrogen of compound **2** with allyl bromide gave compound **3** in an 81% yield. The lactam carbonyl of compound **2** was partially reduced with LiAlH_2_(OEt)_2_ in THF to generate the aminal, [66] which was then treated with KCN and H_2_O to give the α-aminonitrile TM-(–)-52 as a single diastereomer. Chemoselective debenzylation was achieved with BCl_3_ in the presence of pentamethylbenzene to give the phenol. [67] Finally, oxidation of this obtained phenol using salcomine with O_2_ afforded TM-(–)-55 in a 74% yield; whereas compound **2** was bisbenzylated with 2 eq. of benzylbromide in the presence of 10 eq. of NaH to give compound **5** in a 97% yield. The debenzylation of 19 by using BCl_3_ gave the phenol TM-(–)-45. The reductive cyanation of compound **5** generated the aminonitrile compound **6**, which was chemoselectively debenzylated with BCl_3_ to afford TM-(–)-18 in an 89% yield. Finally, the phenol compound **5** was oxidized with salcomine in an oxygen atmosphere to give TM-(–)-4a.

Right-half model of RT compounds in this study namely TM-(–)-45, TM-(–)-18, TM-(–)-4a, TM-(–)-52, and TM-(–)-55 were synthesized as described in Figure 8. TM-(–)-18 and TM-(–)-4a were synthesized according to our previous work [65], whereas TM-(–)-45, TM-(–)-52, and TM-(–)-55 were newly synthesized. All compounds were confirmed to have 99% enantiomeric excess HPLC analysis to prove that there is no racemization at the chiral center in the synthetic route.

#### 4.4.1. Synthesis of (1*R*, 5*S*)-10-(Benzyloxy)-9-methoxy-8, 11-dimethyl-2, 3, 5, 6-tetrahydro-1, 5-epiminobenzo[*d*]azocin-4(1H)-one (compound **2**), (1*R*, 5*S*)-3-Benzyl-10-(benzyloxy)-9-methoxy-8, 11-dimethyl-2, 3, 5, 6-tetrahydro-1, 5-epiminobenzo[*d*]azocin-4(1H)-one (compound **5**), (1*R*, 4*R*, 5*S*)-3-Benzyl-10-(benzyloxy)-9-methoxy-8, 11-dimethyl-1, 2, 3, 4, 5, 6-hexahydro-1, 5-epiminobenzo[*d*]azocine-4-carbonitrile (compound **6**), Synthesis of (1*R*, 4*R*, 5*S*)-3-Benzyl-10-hydroxy-9-methoxy-8, 11-dimethyl-1, 2, 3, 4, 5, 6-hexahydro-1, 5-epiminobenzo[*d*]azocine-4-carbonitrile (TM-(–)-18), and (1*R*, 4*R*, 5*S*)-3-Benzyl-9-methoxy-8, 11-dimethyl-7, 10-dioxo-1, 2, 3, 4, 5, 6, 7, 10-octahydro-1, 5-epiminobenzo[*d*]azocine-4-carbonitrile (TM-(–)-4a)

Asymmetric synthesis methods of compound **2**, compound **5**, compound **6**, (TM-(–)-18), and (TM-(–)-4a) were previously described in the Scheme 4 of Matsubara’s study ‘Asymmetric Synthesis and Cytotoxicity Evaluation of Right-Half Models of Antitumor Renieramycin Marine Natural Products’ [65]. 

#### 4.4.2. Synthesis of (1*R*, 5*S*)-3-Allyl-10-(benzyloxy)-9-methoxy-8, 11-dimethyl-2, 3, 5, 6-tetrahydro-1, 5-epiminobenzo[*d*]azocin-4(1*H*)-one (Compound **3**)

To a solution of lactam compound **2** (10.2 mg, 28.0 µmol) in 1.0 mL dimethylformamide (DMF) was slowly added with sodium hydride (NaH 60% oil dispersion, 2.6 mg, 57.0 µmol, 2.0 eq.) over 10 min at 0 °C. The reaction mixture was stirred at 0 °C for 30 min, after which allyl bromide (AllylBr, 7.0 µL, 57.0 µmol, 2.0 eq.) was added slowly. The mixture was stirred at 25 °C for 14 h. The mixture was diluted with 10 mL water (H_2_O) and extracted with diethyl ether(Et_2_O, 3 × 10 mL). The combined extracts were washed with 10 mL brine, dried over sodium sulfate (Na_2_SO_4_) and concentrated in vacuo to give a residue. The residue was purified by silicon dioxide (SiO_2_) flash column chromatography (CHCl_3_−MeOH = 49:1) to afford compound **3** (9.0 mg, 81%) as a colorless oil. ${\left[\alpha \right]}_{D}^{26}$−72.8 (*c* 0.5, CHCl_3_); ^1^H-NMR (400 MHz, CDCl_3_) δ 7.45–7.32 (5H, m, 10-*O*-Bn-H), 6.70 (1H, s, 7-H), 5.58–5.49 (1H, m, 2′-H), 5.14 (1H, d, *J* = 11.4 Hz, 10-OCH_2_Ph), 5.08 (1H, d, *J* = 11.4 Hz, 10-OCH_2_Ph), 4.92 (1H, dd, *J* = 8.9, 1.4 Hz, 3′-H), 4.62 (1H, dd, *J* = 15.6, 1.4 Hz, 3′-H), 3.99–3.90 (1H, s, 1-H, overlapped), 3.99–3.90 (1H, m, 1′-H), 3.81 (3H, s, 9-OCH_3_), 3.80–3.72 (1H, m, 1′-H), 3.80–3.72 (1H, m, 2-H, overlapped), 3.60 (1H, brd, *J* = 6.4 Hz, 5-H), 3.12 (1H, dd, *J* = 17.1, 6.4 Hz, 6-H), 3.04 (1H, d, *J* = 11.7 Hz, 2-H), 2.78 (1H, d, *J* = 17.1 Hz, 6-H), 2.30 (3H, s, ^11^*N*-CH_3_), 2.26 (3H, s, 8-CH_3_); ^13^C-NMR (100 MHz, CDCl_3_) δ 169.9 (s, C-4), 149.4 (s, C-9), 148.4 (s, C-10), 137.5 (s, Bn), 132.0 (d, C-2′), 131.9 (s, C-8), 131.4 (d, Bn), 128.6 (d × 2, Bn), 128.5 (s, C-6a), 128.2 (d × 2, Bn), 126.3 (s, C-10a), 125.9 (d, C-7), 116.2 (t, C-3′), 74.4 (t, 2′-OCH_2_Ph), 60.1 (q, 9-OCH_3_), 59.3 (d, C-5), 51.5 (d, C-1), 51.0 (t, C-2), 48.0 (t, C-1′), 39.7 (q, ^11^*N*-CH_3_), 27.2 (t, C-6), 15.7 (q, 8-CH_3_); IR (KBr) 3007, 2940, 2340, 1636, 1493, 1337, 1061, 700 cm^−1^; EIMS m/z (%) 392 (M^+^, 28), 301 (12), 295 (23), 294 (100), 204 (36), 203 (49); HREIMS m/z 392.2099 (M^+^, calcd for C_24_H_28_N_2_O_3_, 392.2100).

#### 4.4.3. Synthesis of (1*R*, 4*R*, 5*S*)-3-Allyl-10-(benzyloxy)-9-methoxy-8, 11-dimethyl-1, 2, 3, 4, 5, 6-hexahydro-1, 5-epiminobenzo[*d*]azocine-4-carbonitrile (TM-(–)-52)

To a solution of lactam compound **3** (128 mg, 326 µmol) in 16 mL tetrahydrofuran (THF) at 0 °C was added lithium diethoxyaluminum hydride, (LiAlH_2_(OEt)_2_, 1.0 mol/L in CH_2_Cl_2_, 3.90 mL, 3.90 mmol, 12 eq.) over 10 min. The reaction mixture was stirred for 3 h at 0 °C. The mixture was quenched with acetic acid (AcOH, 390 µL, 6.78 mmol, 20.8 eq.), followed by the addition of potassium cyanide (KCN 129 mg, 1.96 mmol, 6.0 eq.) in H_2_O (2.0 mL), and stirring was continued for 11.5 h at 25 °C. The mixture was neutralized with 5% sodium bicarbonate (NaHCO_3_) solution and diluted with saturated Rochell’s salt aq., and the mixture was stirred for 1.5 h. The mixture was extracted with 3 × 100 mL of chloroform (CHCl_3_). The combined extracts were washed with brine (100 mL), dried over Na_2_SO_4_, and concentrated in vacuo to give a residue. The residue was purified by SiO_2_ flash column chromatography (n-Hex.−EtOAc = 2:1) to afford TM-(–)-52 (70.4 mg, 53%) as a colorless oil. ${\left[\alpha \right]}_{D}^{24}$ −26.9 (*c* 0.2, CHCl_3_); ^1^H-NMR (400 MHz, CDCl_3_) δ 7.41–7.30 (5H, m, 10-*O*-Bn-H), 6.64 (1H, s, 7-H), 5.58–5.48 (1H, m, 2′-H), 5.11–5.02 (2H, m, 3′-H), 5.07 (2H, s, 10-OCH_2_Ph), 3.92 (1H, brs, 1-H), 3.85 (3H, s, 9-OCH_3_), 3.78 (1H, brs, 4-H), 3.25 (1H, brd, *J* = 7.6 Hz, 5-H), 3.05–2.92 (3H, m, 1′-H, 6-H, overlapped), 2.77 (1H, dd, *J* = 11.1, 3.1 Hz, 2-H), 2.57 (1H, d, *J* = 11.1 Hz, 2-H), 2.39 (1H, d, *J* = 17.9 Hz, 6-H), 2.27 (3H, s, 8-CH_3_), 2.12 (3H, s, ^11^*N*-CH_3_); ^13^C-NMR (100 MHz, CDCl_3_) δ 148.9 (s, C-9), 148.3 (s, C-10), 137.5 (s, Bn), 133.5 (d, C-2′), 130.1 (s, C-8), 130.1 (s, C-6a), 128.6 (d × 2, Bn), 128.5 (d × 2, Bn), 128.2 (d, Bn), 126.6 (s, C-10a), 124.4 (d, C-7), 118.8 (t, C-3′), 116.4 (s, 4-CN), 74.5 (t, 10-OCH_2_Ph), 60.0 (q, 9-OCH_3_), 58.8 (d, C-4), 57.9 (t, C-1′), 55.2 (d, C-5), 54.2 (t, C-2), 52.6 (d, C-1), 41.2 (q, ^11^*N*-CH_3_), 25.1 (t, C-6), 15.8 (q, 8-CH_3_); IR (CHCl_3_) 3013, 2938, 2824, 2226, 1416, 1323, 1157, 1063, 1028 cm^−1^; EI-MS *m/z* (%) 403 (M^+^, 1), 295 (28), 294 (100), 243 (12), 204 (24), 203 (21); HREIMS *m/z* 403.2263 (M^+^, calcd for C_25_H_29_N_3_O_2_, 403.2260).

#### 4.4.4. Synthesis of (1*R*, 4*R*, 5*S*)-3-Allyl-10-hydroxy-9-methoxy-8, 11-dimethyl-1, 2, 3, 4, 5, 6-hexahydro-1, 5-epiminobenzo[*d*]azocine-4-carbonitrile (Compound **4**)

To a solution of TM-(–)-52 (60.0 mg, 149 µmol) and pentamethylbenzene (224 mg, 1.49 mmol, 10.0 eq.) in dichloromethane(CH_2_Cl_2_, 10.0 mL) was added boron trichloride (BCl_3_, 1.0 mol/L in CH_2_Cl_2_, 750 µL, 744 µmol, 5.0 eq.) at −78 °C and the reaction mixture was stirred for 2 h. The mixture was diluted with 10.0 mL CH_2_Cl_2_ and quenched at 0 °C with saturated NaHCO_3_ solution. The reaction mixture was extracted with CH_2_Cl_2_ (3 × 20 mL). The combined extracts were dried over Na_2_SO_4_ and concentrated in vacuo to give a residue. The residue was purified by SiO_2_ flash column chromatography (n-Hex.−EtOAc = 2:1) to afford compound **4** (39.5 mg, 85%) as a colorless amorphous. ${\left[\alpha \right]}_{D}^{26}$ −85.8 (*c* 0.6, CHCl_3_); ^1^H-NMR (400 MHz, CDCl_3_) δ 6.42 (1H, s, 7-H), 5.62 (1H, brs, 10-OH), 5.59–5.49 (1H, m, 2′-H), 5.15–5.07 (2H, m, 3′-H), 4.05 (1H, brs, 1-H), 3.81 (1H, brs, 4-H), 3.78 (3H, s, 9-OCH_3_), 3.31 (1H, brd, *J* = 7.8 Hz, 5-H), 3.09–2.97 (3H, m, 1′-H, 6-H, overlapped), 2.85 (1H, dd, *J* = 11.2, 2.9 Hz, 2-H), 2.72 (1H, d, *J* = 11.2 Hz, 2-H), 2.40 (1H, d, *J* = 17.6 Hz, 6-H), 2.34 (3H, s, ^11^*N*-CH_3_), 2.26 (3H, s, 8-CH_3_); ^13^C-NMR (100 MHz, CDCl_3_) δ 145.4 (s, C-10), 142.7 (s, C-9), 133.6 (d, C-2′), 130.6 (s, C-6a), 128.1 (s, C-8), 120.5 (d, C-7), 119.4 (s, C-10a), 118.8 (t, C-3′), 116.5 (s, 4-CN), 60.7 (q, 9-OCH_3_), 58.7 (d, C-4), 57.9 (t, C-1′), 55.3 (d, C-5), 53.5 (t, C-2), 52.3 (d, C-1), 41.5 (q, ^11^*N*-CH_3_), 25.0 (t, C-6), 15.8 (q, 8-CH_3_); IR (CHCl_3_) 3536, 3013, 2940, 2824, 2226, 1458, 1418, 1227, 1157, 1059 cm^−1^; EIMS *m/z* (%) 313 (M^+^, 1), 205 (22), 204 (100); HREIMS *m/z* 313.1789 (M^+^, calcd for C_18_H_23_N_3_O_2_, 313.1790).

#### 4.4.5. Synthesis of (1*R*, 4*R*, 5*S*)-3-Allyl-9-methoxy-8, 11-dimethyl-7, 10-dioxo-1, 2, 3, 4, 5, 6, 7, 10-octahydro-1, 5-epiminobenzo[*d*]azocine-4-carbonitrile (TM-(–)-55)

To a solution of phenol compound **4** (24.0 mg, 76.6 µmol) in 1 mL THF was added salcomine (37.7 mg, 115 µmol, 1.5 eq.) at 25 °C, and the mixture was stirred for 2 h under oxygen (O_2_) atmosphere. The mixture was filtered through a cellulose pad and washed with ethyl acetate (EtOAc). The filtrate was concentrated in vacuo to give a residue. The residue was purified by SiO_2_ flash column chromatography (n-Hex.−EtOAc = 2:1) to afford compound TM-(–)-55 (18.6 mg, 74%) as a yellow oil. ${\left[\alpha \right]}_{D}^{26}$ −41.8 (*c* 0.6, CHCl_3_); ^1^H-NMR (300 MHz, CDCl_3_) δ 5.63–5.52 (1H, m, 2′-H), 5.25 (1H, dd, *J* = 17.2, 1.4 Hz, 3′-H), 5.17 (1H, dd, *J* = 10.3, 1.4 Hz, 3′-H), 4.02 (3H, s, 9-OCH_3_), 3.84 (1H, brs, 1-H), 3.74 (1H, brs, 4-H), 3.33 (1H, brd, *J* = 7.6 Hz, 5-H), 3.09 (1H, ddt, *J* = 13.5, 5.3, 1.4 Hz, 1′-H), 3.02 (1H, dd, *J* = 13.5, 7.6 Hz, 1′-H), 2.84 (1H, dd, *J* = 11.6, 3.2 Hz, 2-H), 2.72 (1H, dd, *J* = 20.8, 7.6 Hz, 6-H), 2.55 (1H, d, *J* = 11.6 Hz, 2-H), 2.32 (3H, s, ^11^*N*-CH_3_), 2.17 (1H, d, *J* = 20.8 Hz, 6-H), 1.97 (3H, s, 8-CH_3_); ^13^C-NMR (100 MHz, CDCl_3_) δ 186.9 (s, C-7), 182.3 (s, C-10), 155.2 (s, C-9), 140.9 (s, C-6a), 137.2 (s, C-10a), 133.0 (d, C-2′), 128.7 (s, C-8), 119.8 (t, C-3′), 115.8 (s, 4-CN), 60.9 (q, 9-OCH_3_), 57.9 (d, C-4), 57.8 (t, C-1′), 54.4 (d, C-5), 51.8 (t, C-2), 51.2 (d, C-1), 41.4 (q, ^11^*N*-CH_3_), 20.8 (t, C-6), 8.7 (q, 8-CH_3_); IR (CHCl_3_) 2936, 2820, 2228, 1659, 1630, 1308, 1236, 1165 cm^−1^; EIMS *m/z* (%) 327 (M^+^, 8), 220 (16), 219 (95), 218 (100), 204 (35), 201 (12), 190 (11), 176 (11); HREIMS *m/z* 327.1582 (M^+^, calcd for C_18_H_21_N_3_O_3_, 327.1583).

#### 4.4.6. Synthesis of (1*R*, 5*S*)-3-Benzyl-10-hydroxy-9-methoxy-8, 11-dimethyl-2, 3, 5, 6-tetrahydro-1, 5-epiminobenzo[*d*]azocin-4(1H)-one (TM-(–)-45)

To a solution of compound **5** (30.8 mg, 67.8 µmol) and pentamethylbenzene (101 mg, 678 µmol, 10.0 eq.) in CH_2_Cl_2_ (10.0 mL) was added BCl_3_ (1.0 mol/L in CH_2_Cl_2_, 340 µL, 339 µmol, 5.0 eq.) and the mixture was stirred for 2 h at −78 °C. The mixture was diluted with 5.0 mL CH_2_Cl_2_ and quenched at 0 °C with saturated NaHCO_3_ solution. The reaction mixture was extracted with CH_2_Cl_2_ (3 × 10 mL). The combined extracts were dried over Na_2_SO_4_ and concentrated in vacuo to give a residue. The residue was purified by SiO_2_ flash column chromatography (CHCl_3_−MeOH = 9:1) to afford TM-(–)-45 (14.3 mg, 89%) as a colorless amorphous. ${\left[\alpha \right]}_{D}^{27}$ −290.7 (*c* 0.8, CHCl_3_); ^1^H-NMR (400 MHz, CDCl_3_) δ 7.15–7.06 (3H, m, ^3^*N*-Bn-H), 6.82–6.80 (2H, m, ^3^*N*-Bn-H) 6.53 (1H, s, 7-H), 4.69 (1H, d, *J* = 15.0 Hz, ^3^*N*-CH_2_Ph), 4.35 (1H, d, *J* = 15.0 Hz, ^3^*N*-CH_2_Ph), 4.14 (1H, brd, *J* = 4.6 Hz, 1-H), 3.78 (1H, dd, *J* = 11.9, 4.6 Hz, 2-H), 3.70 (1H, d, *J* = 6.4 Hz, 5-H), 3.68 (3H, s, 9-OCH_3_), 3.19 (1H, dd, *J* = 17.0, 6.4 Hz, 6-H), 3.10 (1H, d, *J* = 11.9 Hz, 2-H), 2.87 (1H, d, *J* = 17.0 Hz, 6-H), 2.49 (3H, s, ^11^*N*-CH_3_), 2.28 (3H, s, 8-CH_3_); ^13^C-NMR (100 MHz, CDCl_3_) δ 170.4 (s, C-4), 145.5 (s, C-10), 143.4 (s, C-9), 136.4 (s, C-1′), 129.2 (s, C-8), 129.0 (s, C-6a), 128.3 (d × 2, C-3′, C-5′), 127.2 (d × 2, C-2′, C-6′), 126.9 (d, C-4′), 121.8 (d, C-7), 119.2 (s, C-10a), 60.6 (q, 9-OCH_3_), 59.5 (d, C-5), 51.1 (d, C-1), 49.6 (t, C-2), 49.0 (t, ^3^*N*-CH_2_Ph), 40.0 (q, ^11^*N*-CH_3_), 27.9 (t, C-6), 15.7 (q, 8-CH_3_); IR (CHCl_3_) 3528, 3005, 2934, 2359, 1636, 1495, 1233, 1057, 696 cm^−1^; EIMS *m/z* (%) 352 (M^+^, 14), 205 (19), 204 (100), 189 (12); HREIMS *m/z* 352.1788 (M^+^, calcd for C_21_H_24_N_2_O_3_, 352.1787).

### 4.5. Simplified Right-half Model of RT Compounds Preparation

The compounds in solid form were dissolve in DMSO to 50 mM stock solution and stored at −20 °C. They were freshly diluted to concentrations used in the experiments with an awareness that final concentration of DMSO should be less than 0.5%.

### 4.6. Reagents and Antibodies

Roswell Park Memorial Institute (RPMI) 1640 medium, Dulbecco’s Modified Eagle’s Medium (DMEM) medium, penicillin/streptomycin, fetal bovine serum (FBS), phosphate-buffered saline (PBS), L-glutamine, and trypsin-EDTA were purchased from Gibco (Grand Island, NY, USA). Dimethyl sulfoxide (DMSO), 3-(4,5-dimethylthiazol-2-yl)-2,5-diphenyltetrazoliumbromide (MTT), propidium iodide (PI), Hoechst 33342, cisplatin, etoposide, doxorubicin, and bovine serum albumin (BSA) were purchased from Sigma-Aldrich, Co. (St. Louis, MO, USA). The primary antibodies used in the experiments were β-Actin (13E5) rabbit mAb (Cat#4970, RRID: AB_2223172), PARP (46D11) rabbit mAb (Cat# 9532, RRID: AB_2160739), Mcl-1 (D2W9E) rabbit mAb (Cat#94296, RRID: AB_2722740), Bcl-2 (D55G8) rabbit mAb (Cat# 4223, RRID: AB_1903909), and Bax (D2E11) rabbit mAb (Cat#5023, RRID: AB_10557411) which were obtained from Cell Signaling Technology (Danvers, MA, USA). The respective secondary antibodies, anti-rabbit IgG, HRP-linked antibody (Cat#7074, RRID: AB_2099233) was also purchased from Cell Signaling Technology (Danvers, MA, USA). Moreover, goat anti-rabbit IgG H&L (Alexa Fluor 488, Cat#ab150077, RRID: AB_2630356) was obtained from Abcam (Cambridge, MA, USA).

### 4.7. Cell Viability Assay

NSCLC cell line (H460) and patient-derived primary lung cancer cell lines (ELC12, ELC16, ELC17, and ELC20) were seeded in 96-well plates at the density of 1 × 10^5^ cells/well. After incubating overnight, they were treated with various concentrations of chemotherapeutic drugs (cisplatin, etoposide, and doxorubicin), RT, and right-half model of RT compounds (TM-(–)-45, TM-(–)-18, TM-(–)-4a, TM-(–)-52, and TM-(–)-55) for 24 h. Then, 100 μL per well of MTT solution was added to achieve a final concentration of 400 μg/mL and incubated for an additional 3 h at 37 °C. Yellow supernatants of MTT were removed and replaced with 100 μL of DMSO to dissolve formazan crystals. The quantity of formazan product proportional to the number of viable cells was measured by recording change in absorbance at 570 nm by a microplate reader (Anthros, Durham, NC, USA). The percentage of cell viability and IC_50_ were determined as described in the manufacturer’s protocol (7sea Biotech). Cell viability = (ODexperiment − ODblank) / (ODcontrol − ODblank) × 100%. 

### 4.8. Nuclear Staining Assay

Hoechst 33342 and PI double staining were applied to define apoptotic and necrotic cell death through nuclear co-staining. NSCLC cell lines (H460, H292, H23, and A549) and patient-derived primary lung cancer cell lines (ELC12, ELC16, ELC17, and ELC20) were seeded into 96-well plates at the density of 1 × 10^5^ cells/well overnight and treated with 5 and 10 µM of chemotherapeutic agents (cisplatin, etoposide, and doxorubicin), RT, and right-half model of RT compounds (TM-(–)-18 and TM-(–)-4a) for 24 h. Afterwards, the cells were stained with 10 μg/mL Hoechst 33342 for 15 min at 37 °C and then stained with 5 μg/mL PI before immediately detecting fluorescence of nuclear-stained cells by fluorescent microscope (Nikon ECLIPSE Ts2, Tokyo, Japan). The number of nuclear condensed and DNA fragmented cells was reported as the percentage of apoptotic cells.

### 4.9. Immunofluorescence for Mcl-1

Immunofluorescence was introduced to evaluate whether or not cells in particular samples express Mcl-1 through antibody specification. NSCLC cell line (H460) and patient-derived primary lung cancer cell lines (ELC12 and ELC16) were seed overnight in 96-well plates at the density of 1 × 10^5^ cells/well. Then, they were treated with 1 µM of RT, TM-(–)-18, and TM-(–)-4a and incubated for 24 h. After that, cells were fixed with 4% of paraformaldehyde for 30 min, permeabilized by 0.5% of Triton X-100 in PBS for 5 min, followed by blocking with 10% of FBS in 0.1% of Triton X-100 for further 1 h at room temperature (RT). Primary antibody of Mcl-1 at proportional 1:100 in 10% of FBS was applied to the cells before incubation overnight at 4 °C. After incubation time, Alexa Fluor 488 conjugated with goat anti-rabbit IgG secondary antibody was added and incubated in dark for 1 h at RT. Cell nucleuses were stained with Hoechst 33342 and then visualized under fluorescent microscope (Nikon ECLIPSE Ts2, Tokyo, Japan).

### 4.10. Western Blot Analysis

Western blot analysis was used to determine the amount of specific proteins in the cells. NSCLC cell line (H460) and patient-derived primary lung cancer cell lines (ELC12, ELC16, ELC17, and ELC20) were seeded overnight at the density of 4×10^5^ cells/well and treated with RT, TM-(–)-45, TM-(–)-18, TM-(–)-4a, TM-(–)-52, and TM-(–)-55) for 24 h. Cells were then collected by centrifuging media with 1500 rpm for 5 min and lysed with radioimmunoprecipitation assay (RIPA) lysis buffer containing 25 mM Tris-HCl pH 7.6, 150 mM NaCl, 1% NP-40, 1% sodium deoxycholate, 0.1% SDS, and a protease inhibitor cocktail (Roche Diagnostics, Indianapolis, IN, USA) for 30 min at 4 °C. The lysates were collected and their protein contents were determined by a BCA protein assay kit (Pierce Biotechnology, Rockford, IL, USA). Equivalent amount of proteins from each sample (70 μg) was separated using SDS-polyacrylamide gel electrophoresis and further transferred to 0.2 μm polyvinylidene difluoride (PVDF) membranes (Bio-Rad Laboratories, Hercules, CA, USA). 5% skim milk in TBST (Tris-buffer saline with 0.1% tween containing 25 mM Tris-HCl pH 7.5, 125 mM NaCl and 0.1% tween 20) was applied to block the separating blots for 2 h at RT. Membranes were incubated with primary antibodies specific for PARP, Mcl-1, Bcl-2, Bax, and β-Actin at 4 °C overnight, washed with TBST and then were incubated with secondary antibody for 2 h at RT. Finally, the immunoreactive proteins were detected with the enhanced chemiluminescent detection system (Supersignal West Pico, Pierce, Rockford, IL, USA) and subsequently exposed to X-ray film. The intensity of protein bands was analyzed by the ImageJ software (version 1.52, National Institutes of Health, Bethesda, MD, USA). Densitometric values of protein expression levels were calculated as the fold changes relative to β-actin. Detailed information can be found at Supplementary Figures S1–S3.

### 4.11. Computational Mcl-1 modelling and Molecular Docking

Molecular docking was applied to detect an interaction between RT or simplified right-half model of RT compounds and Mcl-1 protein. The target sequence of Mcl-1 (350 amino acids) was retrieved from UniProt, accession code Q07820 [68]. Since the three-dimensional (3D) structure of Mcl-1 has not been available yet, homology modelling, a promising tool to predict 3D structure of protein, was performed using the I-TASSER server [69,70]. The quality of the constructed homology model was then estimated by the Ramachandran plot using PROCHECK server [71]. The information in Figure 6b showed that the majority of amino acids were mainly found in most favored and additional allowed regions with values of 77.8% and 17.6%, respectively, which is suggestive of a reliable quality of this model. Before docking calculations, the modeled structure of Mcl-1 was relaxed by short molecular dynamics simulation for 20 ns using the AMBER16 software package according to standard procedures [72,73,74,75].

To prepare for the docking study, the chemical structures of all studied ligands were built using the Gaussian09 program [76]. Afterward, quantum chemistry calculation with the B3LYP/6-31G* level of theory was used for geometry optimization of all compounds. The docking calculations were carried out with AutoDock Vina [77]. Each ligand was docked to the expected binding site of Mcl-1 (residues 137–143). A grid box size was set to of 20 × 20 × 20 Å, whereas the grid was centered at the position of the residues 137–143, at x, y, z coordinates of 54.0, 31.0, 51.5. The UCSF Chimera package [78] was used for the graphical presentation of the data.

### 4.12. Statistical Analysis

The data from at least three independent replicated experiments (*n* = 3) was presented as the mean ± standard error of the mean (SEM). Statistical differences between multiple groups were analyzed using an analysis of variance (ANOVA) which calculated SPSS software program version 16 (SPSS Inc., Chicago, IL, USA). Statistical significance was considered at *p* < 0.05.

## 5. Conclusions

This study provides supporting evidence that RT and its simplified right-half model compounds TM-(–)-18 and TM-(–)-4a exert an anti-cancer action through Mcl-1 suppression and in part by the decrease in Bcl-2. Furthermore, by synthesizing structurally modified compounds as analogues of RT, and by performing in vitro protein analysis and molecular docking experiments, we were able to clarify the SAR information of RT compounds, which indicated that the cyanide and benzene ring compositions of RT play key functions in targeting the Mcl-1 protein. RT, TM-(–)-18, and TM-(–)-4a were the active compounds that were demonstrated to have potent anti-cancer activity in lung cancer cells, and information on the SARs of these compounds could encourage the development of related compounds having these groups for Mcl-1 suppression.

## Supplementary Materials

The following are available online at https://www.mdpi.com/2072-6694/12/4/875/s1, Figure S1: The whole western blots of Figure 1; Figure S2 The whole western blots of Figure 3; Figure S3 The whole western blots of Figure 4.
