# Supplementary material for: Structure–Activity Relationships and Molecular Docking Analysis of Mcl-1 Targeting Renieramycin T Analogues in Patient-derived Lung Cancer Cells
Source: Cancers (Basel). 2020 Apr 3;12(4):875. doi: 10.3390/cancers12040875 (PMC7226000; doi:10.3390/cancers12040875)
Supplement: Supplementary file 1 [file cancers-12-00875-s001.pdf]

Figure 1 Supplemental g

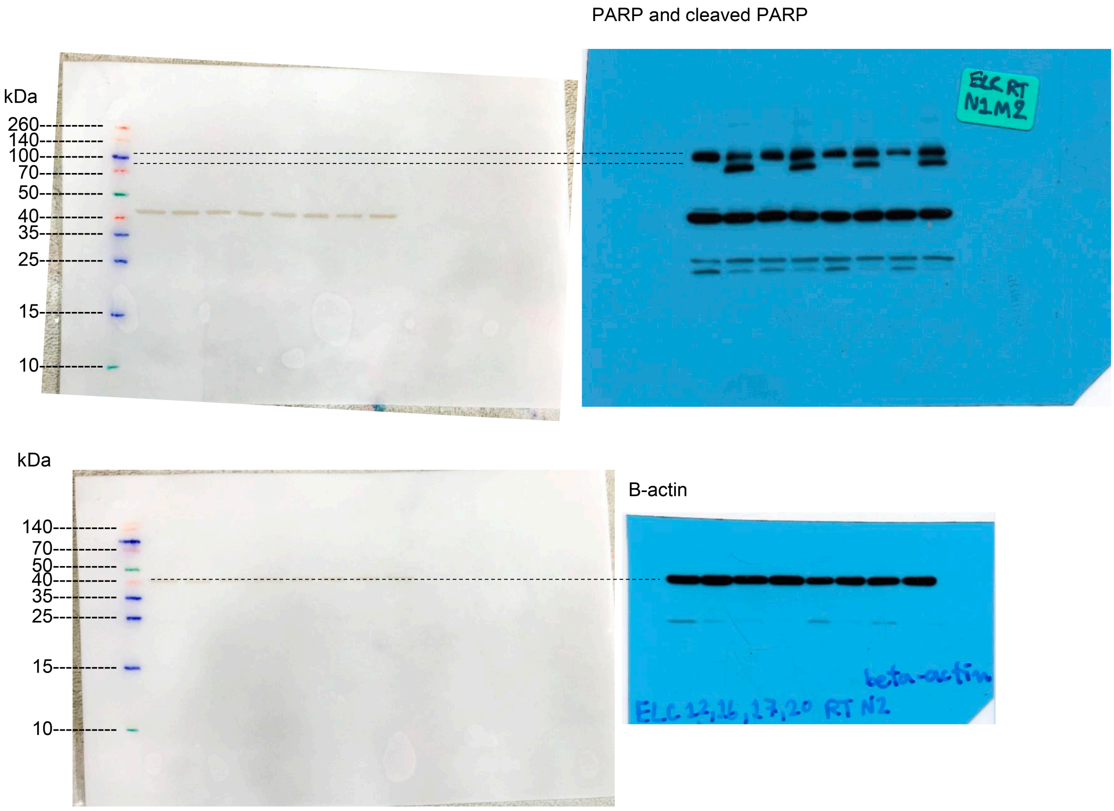

Figure S1. The whole western blots of Figure 1.

a **Figure 3 Supplemental b**

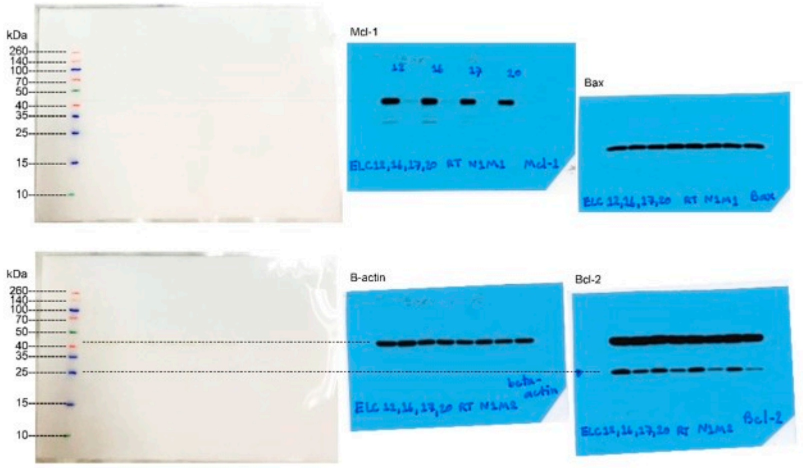

b **Figure 3 Supplemental c**

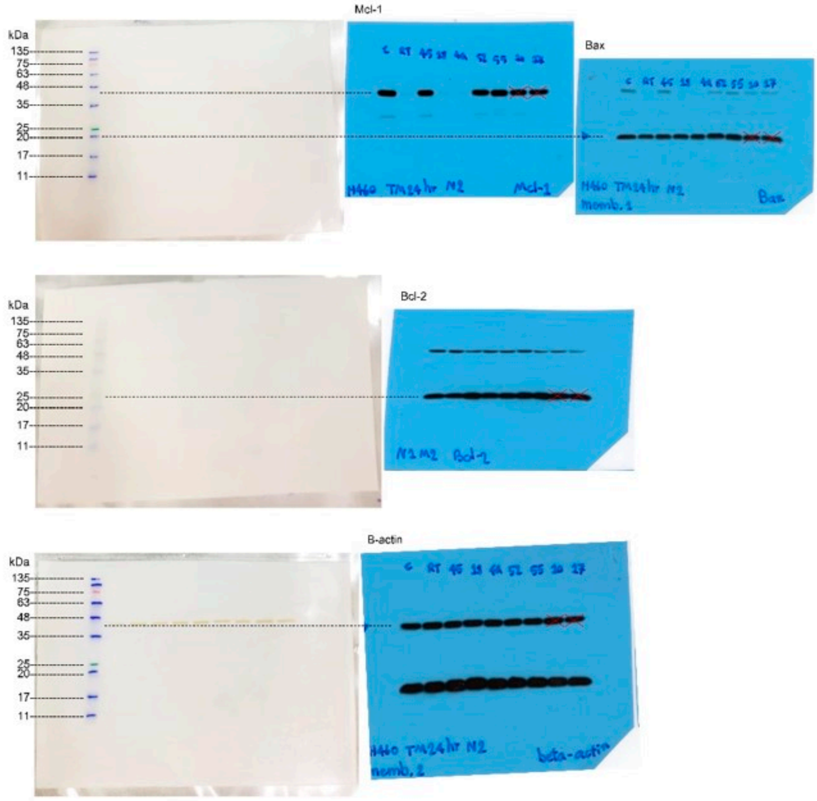

Figure S2 The whole western blots of Figure 3.

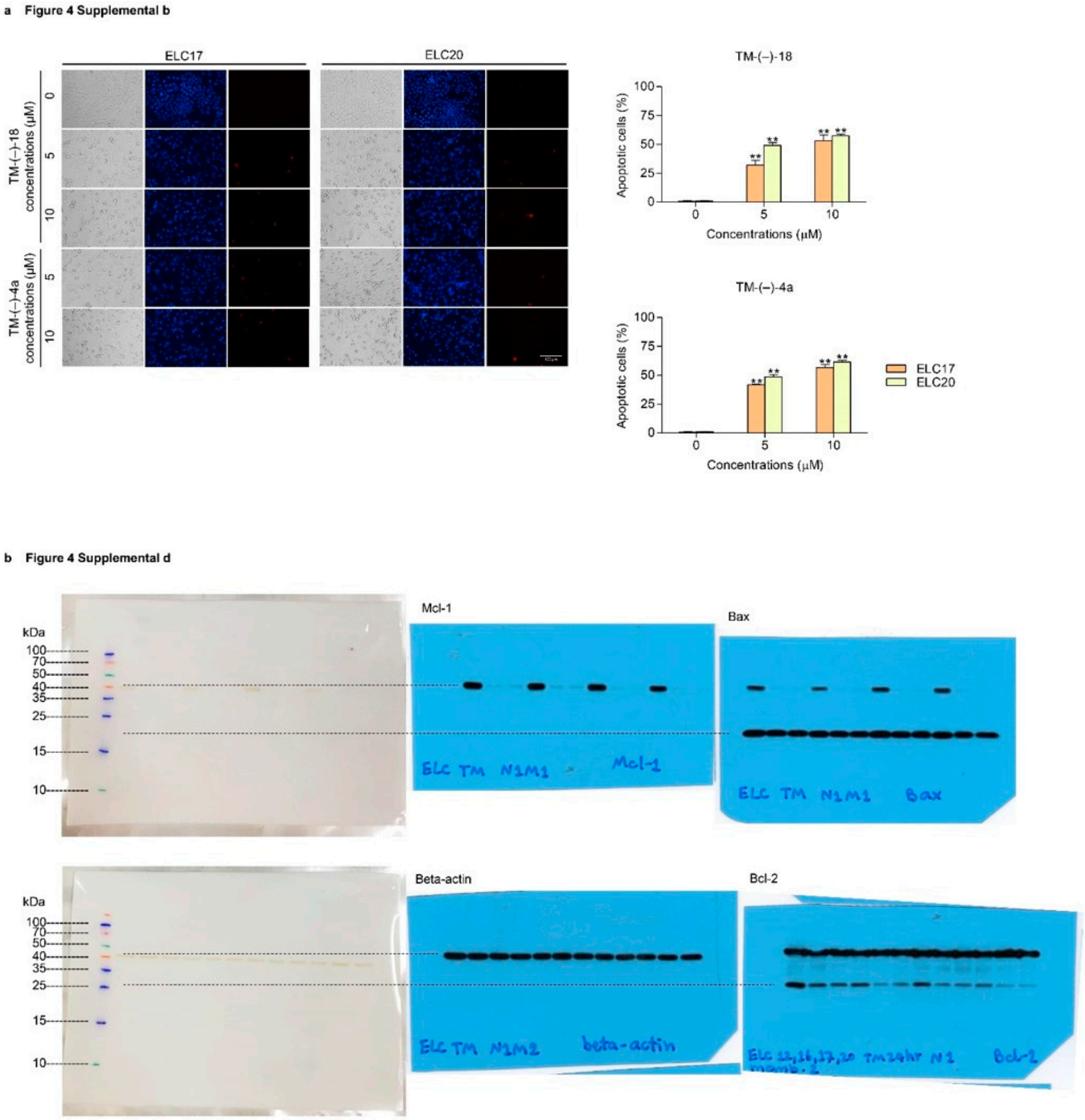

Figure S3 The whole western blots of Figure 4.
